# Harnessing the Health and Techno-Functional Potential of Lactic Acid Bacteria: A Comprehensive Review

**DOI:** 10.3390/foods13101538

**Published:** 2024-05-15

**Authors:** Lamia Ayed, Sana M’hir, Domenico Nuzzolese, Raffaella Di Cagno, Pasquale Filannino

**Affiliations:** 1 Laboratory of Microbial Ecology and Technology (LETMI), LR05ES08, National Institute of Applied Sciences and Technology (INSAT), University of Carthage, BP 676, Tunis 1080, Tunisia; sana2617@yahoo.fr; 2 Department of Animal Biotechnology, Higher Institute of Biotechnology of Beja, University of Jendouba, BP 382, Beja 9000, Tunisia; 3 Department of Soil, Plant and Food Science, University of Bari Aldo Moro, 70126 Bari, Italy; domenico.nuzzolese1@uniba.it (D.N.); pasquale.filannino1@uniba.it (P.F.); 4 Faculty of Agricultural, Environmental and Food Sciences, Libera Università di Bolzano, 39100 Bolzano, Italy; raffaella.dicagno@unibz.it

**Keywords:** lactic acid bacteria, probiotic, bioactive compounds, techno-functional proprieties, health benefits

## Abstract

This review examines the techno-functional properties of lactic acid bacteria (LABs) in the food industry, focusing on their potential health benefits. We discuss current findings related to the techno-functionality of LAB, which includes acidification, proteolytic and lipolytic features, and a variety of other biochemical activities. These activities include the production of antimicrobial compounds and the synthesis of exopolysaccharides that improve food safety and consumer sensory experience. LABs are also known for their antioxidant abilities, which help reduce oxidative reactions in foods and improve their functional properties. In addition, LABs’ role as probiotics is known for their promising effects on gut health, immune system modulation, cholesterol control, and general wellbeing. Despite these advantages, several challenges hinder the effective production and use of probiotic LABs, such as maintaining strain viability during storage and transport as well as ensuring their efficacy in the gastrointestinal tract. Our review identifies these critical barriers and suggests avenues for future research.

## 1. Introduction

Lactic acid bacteria (LABs) are important active agents in the food industry and are best known for their role in the lactic acid fermentation process. This process not only acidifies the product, but also increases the bioavailability of essential nutrients, such as vitamins, minerals, and antioxidant molecules, allowing the body to better absorb nutrients and bioactive compounds [1]. Additionally, LABs contribute substantially to the desirable texture, flavor, and aroma of fermented foods, significantly enriching both the sensory experience and the nutritional value of these products [1]. Moreover, employing LABs is an effective natural method for food preservation, extending shelf life and maintaining food properties. This is achieved through the production of lactic acid and other microbial metabolites, including bacteriocins and hydrogen peroxide, which inhibit the growth of spoilage organisms and pathogens [2].

In addition to their technological advantages, LABs are also highly valued for their probiotic properties. They play a crucial role in promoting gut health and supporting digestion by maintaining a balanced gut microbiota, which is essential for a robust gut immune system. Probiotic LABs work by displacing harmful bacteria, inhibiting their growth and improving the integrity of the intestinal barrier [3,4]. Current research has expanded to explore the relationship between dysbiosis, which is characterized by an imbalanced gut microbiota and inflammation. Such imbalances are considered both contributory to and a consequence of various health issues, linking to symptoms like fatigue, diarrhea, bloating, digestive problems, insulin resistance, and immune deficiencies [5]. Probiotics have also been shown to prevent ailments, such as food allergies, allergic rhinitis, and gastrointestinal disorders, including necrotizing enterocolitis [6,7]. Given the increasing consumer demand for healthful and sustainable food options, the incorporation of probiotic LABs and their metabolites into food products is gaining traction. This growing interest underscores the need for ongoing research into new applications for LABs, focusing on enhancing overall food quality and human health.

This review embarks on an exploration of the techno-functional properties of LABs in food products, paying close attention to the potential benefits of LABs, as well as their significance in relation to human wellbeing. Furthermore, this review addresses the challenges associated with the production and application of probiotic LABs and proposes new directions for future research.

## 2. Lactic Acid Bacteria Classification

LABs form a large group of Gram-positive, non-sporulating, rod- or cocci-shaped bacteria. Among LABs, lactobacilli are a relevant subgroup with 256 species that have high technological relevance. Due to their high diversity at phenotypic, ecological, and genotypic levels, lactobacilli have recently been reclassified into 25 genera, including the emended genus *Lactobacillus*, *Paralactobacillus*, and 23 novel genera. Other relevant LABs are grouped into *Lactococcus*, *Leuconostoc*, *Pediococcus*, *Streptococcus*, *Aerococcus*, *Alloiococcus*, *Carnobacterium*, *Dolosigranulum*, *Enterococcus*, *Oenococcus*, *Tetragenococcus*, *Vagococcus*, and *Weissella* genera [8,9]. They are facultatively anaerobic, catalase-negative, and stationary. They can be found in a range of habitats, including the cavities of people and animals as well as in ecological niches in dairy, meat, and vegetable products [10,11]. The LAB group is currently classified within the phylum Firmicutes, class Bacilli, and order *Lactobacillales* [11]. These microorganisms are characterized by low GC content (31–49%). While these bacteria display diverse metabolic capacities, their common trait is the production of lactic acid from the provided carbon substrate. They are classified as either obligate homofermentative, predominantly producing lactic acid, or obligate heterofermentative, yielding a variety of metabolites including lactic acid, acetic acid, ethanol, and carbon dioxide. Furthermore, some species are classified as facultative heterofermentative, capable of utilizing both fermentation pathways [11].

### Probiotics

**Figure 1:**
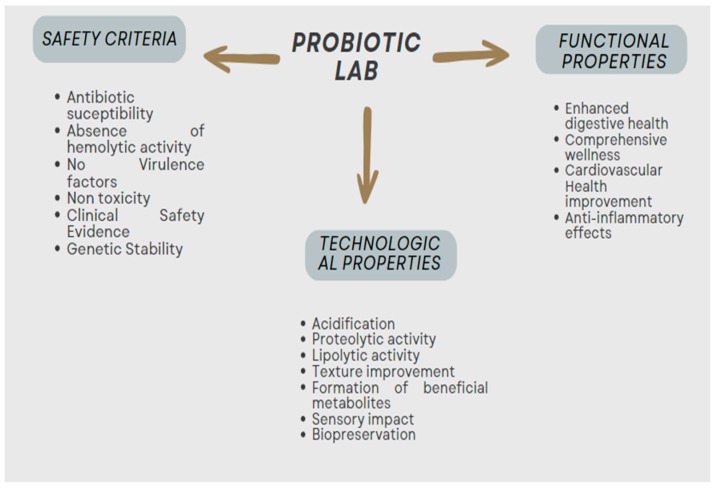
Comprehensive overview of safety, techno-functional, and functional properties of probiotic LABs.

In recent years, there has been an increase in the use of LABs as probiotics, which are living bacteria that, when taken in adequate quantities, have positive effects on the body [12]. Probiotics should be able to tolerate the conditions of the gastrointestinal tract, including the production of bile in the duodenum and the acidic environment of the stomach, and they need to adhere to the gut lining to enhance interactions with gut cells [13]. They must also be able to participate in gut biological activity. Probiotics exert their effects through three main modes of action: modulating the host’s defenses, including the immune system; directly affecting other microorganisms, which is beneficial for preventing infections and restoring microbial balance in the gut; and impacting microbial products, like toxins and bile salts, aiding in detoxification in the gastrointestinal tract [13]. To experience health advantages, a daily intake of 10^8^ to 10^11^ CFU of bacterial cells is recommended [14]. It is crucial to note that while the viability of probiotics is important, non-viable forms, known as parabiotics, can also contribute to human health [15]. In order to establish the functional criteria for probiotics (Figure 1), it is essential to conduct both in vitro and in vivo assays and to validate the results through controlled human studies.

## 3. Safety of LABs

LABs are generally recognized as GRAS (Generally Recognized as Safe), which officially authorizes their use in food applications and guarantees their safety. However, not all LAB strains are classified as GRAS. To achieve GRAS status, a LAB strain must fulfill specific criteria, such as being non-pathogenic, non-mutagenic, non-carcinogenic, and not resistant to antibiotics [11]. Similarly, the European Union, alongside the GRAS designation in the United States, offers a corresponding status known as the Qualified Presumption of Safety (QPS), which was established by the European Food Safety Authority (EFSA). Microorganisms under QPS are recognized as safe without the need for further comprehensive safety assessments. To qualify for QPS status, a microorganism must have a well-defined taxonomic identity, adequate evidence of safety, confirmed non-pathogenic properties, and a clearly defined intended use [12]. The Generally Recognized as Safe (GRAS) classification is primarily assigned to *Lactococcus* and lactobacilli. Certain species within LAB genera such as *Streptococcus* have also received GRAS/QPS designation; however, none of the species within the *Enterococcus* genus have achieved GRAS/QPS status so far, mainly due to their potential as opportunistic pathogens [13].

## 4. Techno-Functional Proprieties of LABs

LABs exert a significant influence on the technological and functional characteristics of food products. These characteristics, which include safety assurance, nutritional enrichment, and sensory enhancement, underscore the essential role of LABs in preserving and improving food quality. Understanding the complex techno-functional characteristics of LABs is pivotal for optimizing food manufacturing procedures and guaranteeing customer satisfaction.

### 4.1. Acidification

Their acidifying activity has a significant impact on the functional properties of fermented foods and beverages. The decrease in pH plays an important role in improving their hygienic qualities and imparting distinct taste profiles. Several factors such as fermentation temperature, strain, and the nature and concentration of the carbon source can affect lactic acid production [16]. For example, lactose remains the preferred carbon source for some LABs. Grasso et al. [17] found that dairy yogurt had higher total titratable acidity and L-lactic acid content compared to plant-based yogurts.

Furthermore, acidification creates an acidic environment that is conducive to the coagulation of casein in dairy products and enhances water retention capacity and moisture retention during the processing and cooking of meat products [18,19]. Barbut’s research [20] identified that the gelation process in meat products is significantly influenced by the timing of acid exposure to meat proteins. Specifically, lactic acid plays a crucial role by facilitating the binding of these proteins before the cooking process. This pre-cooking binding is essential for forming a stable gel structure, which improves the texture of the meat. Additionally, maintaining a low pH not only enhances the color and stability of meat products but also improves the color and quality of fermented fruit beverages [21,22].

In addition to enhancing the techno-functional properties of foods, acidification also improves their nutritional properties. Notably, it improves the solubilization and extraction of bioactive compounds in fermented fruit beverages [23]. Several studies have shown that the fermentation of fruit juices significantly affects the levels of citric acid, the biochemical conversion of phenolic compounds, and antioxidant activity [22,24]. However, LABs can also be responsible for biological deacidification, as is the case in winemaking, where LABs convert malic acid into lactic acid, thus softening the taste [25]. An improvement in the nutritional value of sourdough bread was also noted, including improved mineral availability and a reduction in phytic acid content due to lactic acid production [26].

LABs can also produce beneficial organic acids, like acetate and propionate, which are known for improving food preservation by reducing the pH and enhancing sensory qualities, such as flavor and texture, in products like cheese and yogurt [27]. These acids also support overall health, including digestive and metabolic processes. Additionally, compounds, like 2-hydroxyisocaproic acid (HICA) and 3-phenyllactic acid (PLA), which are derived from the amino acids L-leucine and L-phenylalanine, respectively, possess antimicrobial properties that help extend the shelf life of food products while contributing to health benefits, such as muscle recovery and metabolic health [28].

### 4.2. Protein Hydrolysis

The significance of the proteolytic activities of LABs in the food industry is well recognized. Thermophilic lactobacilli are particularly known for their high proteolytic activity, surpassing that of other bacteria, with variations among strains within each species [29]. Among them, *Lactobacillus delbrueckii* subsp. *bulgaricus* stands out for its strong proteolytic capacity [30]. This activity involves a range of proteinases and peptidases, which are essential for flavor development and the production of various bioactive compounds, including antimicrobial and antioxidant peptides [31].

These proteolytic activities contribute to the coagulation of milk proteins, influencing the texture and taste of the dairy products [32]. Moreover, they enhance the safety of fermented products by reducing allergenic properties through the hydrolysis and elimination of protein allergens [33]. In fermented meat products, LAB proteolytic activity aids in the breakdown of proteins—thereby improving digestibility and increasing the number of soluble and free amino acids—and small peptides [33]. This process not only enhances palatability and tenderizes the meat but also reduces the required maturation period [34].

In the bakery industry, proteolytic activity is crucial for breaking down gluten proteins, thus affecting the rheology of sourdough wheat dough and impacting bread texture and digestibility [26]. Additionally, proteolytic activity leads to the release of various amino acids in fermented products, including glutamate, which enhances the umami taste, and peptides such as glutathione and glutamyl dipeptides, contributing to the kokumi taste [35,36].

### 4.3. Impact of LABs on Food Texture

LABs significantly influence the texture of food products primarily through their exopolysaccharide (EPS) production [37]. EPSs act as natural texturizers, stabilizers, viscosifiers, bio-thickeners, or emulsifiers [38]. The specific function of EPSs depends on factors such as temperature, pH, ionic strength of the medium, sugar composition, chain length, sugar linkages, branching frequency, and molecular mass [38]. In dairy products, EPSs enhance texture by contributing to a smoother, creamier consistency and by improving the body of low-fat cheeses without the need for added fats [38]. They also modify the rheological properties of food matrices, leading to increased viscosity and improved gel strength, which are beneficial for products like fermented dairy drinks and cheeses [39,40]. Additionally, EPSs help reduce syneresis in yogurt and other fermented milk products, thus preventing the undesirable separation of liquid from the gel [40].

EPSs also have excellent water-binding capacities, which are essential for retaining moisture in baking products. This property helps reduce staling and improves the softness and shelf life of bread [37]. Galli et al. [41] observed that fermentation using *Weissella confusa* results in the in situ production of dextran, which increases dough viscosity. Furthermore, recent studies suggest that strains of *Limosilactobacillus reuteri*-producing EPSs can enhance the quality of gluten-free bread, reducing the need for expensive hydrocolloidal polysaccharides in the baking process [39].

### 4.4. Production of Flavor Compounds

During food fermentation, LABs utilize carbon sources, initiating specific metabolic pathways that significantly enhance flavor profiles. These bacteria primarily ferment carbohydrates into lactic acid, which not only preserves the food by lowering its pH but also imparts a distinctive tartness. During this process, LABs also generate various volatile compounds, such as alcohols, esters, and aldehydes, which are essential for the nuanced flavors found in cheeses, yogurts, and sourdough [42,43]. LABs can also convert citric acid into diacetyl and acetoin, which contribute buttery and creamy flavors, respectively, in dairy products [44,45].

LABs also contribute to flavor development in fermented foods through their lipid metabolism. They initiate lipolysis, breaking down triglycerides into free fatty acids and glycerol [46]. Release of short-chain fatty acids, such as butyric and caproic acid, are essential for the distinctive flavors found in cheeses and other dairy products [47,48].

LABs’ ability to metabolize amino acids also plays an important role in flavor diversification [49]. LABs can decarboxylate or transaminate amino acids, converting them into α-keto acids and amines, which serve as precursors for various aromatic compounds. These α-keto acids may be further reduced into aldehydes and alcohols, contributing significantly to the flavor profile of fermented foods [50]. This enzymatic breakdown enhances the flavor profiles of fermented products, adding depth and richness. Additionally, amino acids can participate in Maillard reactions with reducing sugars present in the fermentation medium, leading to the formation of complex flavors, adding characteristics (like umami and caramel) and reducing undesirable odors [51]. Alcohols derived from amino acids can also react with free fatty acids to form esters, which are known for their pleasant fruity and floral aromas [47,52].

### 4.5. Improvement of Antioxidant Activity of Fermented Products

LABs enhance the antioxidant activity of fermented products through multiple biochemical processes. During fermentation, these bacteria promote the release of antioxidant compounds, such as free phenolic compounds, which directly contribute to terminating oxidation chain reactions [53]. Research has shown that changes in phenolic compounds during lactic acid fermentation are associated with increased antioxidant levels [23]. Additionally, specific enzymes like tannase and β-glucosidase in LABs break down tannins and glycosylated isoflavones, respectively. This process releases aglycones and polyphenol monomers, enhancing the bioavailability and antioxidant activity of these substances [23,54,55].

Furthermore, in protein-rich foods, LABs play a crucial role in converting proteins into smaller peptides and free amino acids, which have significant antioxidant activities that can neutralize reactive oxygen species. Research has demonstrated that the proteolytic activities of LAB strains, like *Lacticaseibacillus casei* and *Lactobacillus acidophilus*, in probiotic yogurts and fermented milk, enhance the antioxidant capacity. This enhancement occurs as antioxidant peptides are released during proteolysis [56]. These peptides, noted for their potent antioxidant properties, function by chelating pro-oxidant metal ions. Their effectiveness is determined by their structural features and concentration levels [57,58].

Additionally, LABs produce EPSs with intrinsic antioxidant properties that further enhance free radical scavenging. An EPS isolated from a *Lactiplantibacillus plantarum* culture was found to exhibit antioxidant activity by acting as an electron donor to stabilize free radicals, among other not yet fully understood mechanisms [59].

### 4.6. Biopreservation

LABs play a crucial role in food biopreservation due to their ability to produce a wide range of antifungal and antibacterial metabolites [60,61]. These include organic acids, such as lactic acid and acetic acid, which lower the pH of the food environment, inhibiting the growth of molds like *Penicillium* species as well as common foodborne pathogens [62,63].

LABs also produce hydrogen peroxide a potent oxidizing agent, is known for its ability to damage cellular components, such as biofilms, cell membranes, and cell walls, offering a broad antimicrobial range against bacteria, fungi, and viruses [64]. This property makes it ideal for disinfection and food preservation. Additionally, LABs produce volatile compounds, like diacetyl, which not only contribute to the aroma of cheeses but also inhibit the growth of molds [65]. Moreover, LABs produce conjugated linoleic acid (CLA), which is crucial in boosting the effectiveness of LABs against pathogenic bacteria [66].

EPSs produced by LABs also play a crucial role in disrupting the biofilms of pathogenic bacteria, thereby enhancing food safety [67]. These substances interfere with the initial stages of biofilm development by altering the surfaces of bacterial cells, which prevents their attachment to surface [68]. Moreover, EPSs can disrupt the communication among pathogenic bacteria by interfering with quorum sensing mechanisms, which are crucial for biofilm maturation and integrity [69]. Some EPSs also possess inherent antimicrobial properties that directly inhibit or kill pathogens within biofilms [67]. The *Lactococcus lactis F-mou* strain, isolated from Sahrawi camel milk in Algeria, produces EPSs with potent inhibitory properties against various harmful microorganisms, including *Staphylococcus aureus*, *Pseudomonas aeruginosa*, *Escherichia coli*, *Listeria monocytogenes*, and others, showcasing its significant potential in natural food preservation and enhancement [70].

Bacteriocins, which are small antimicrobial peptides produced by LABs, are highly effective at inhibiting a wide range of spoilage and pathogenic microorganisms, thus enhancing food safety and extending shelf life [71,72]. Recognized as safe for human consumption, bacteriocins offer a natural alternative to chemical preservatives in the food industry. Nisin, a well-known bacteriocin produced by *Lactococcus lactis* subsp. *lactis*, has been classified as GRAS and deemed a safe food additive since 1969 [73]. It is widely used in the dairy industry to inhibit spoilage and pathogenic bacteria in products like cheese and yogurt. Nisin is particularly effective against Gram-positive bacteria, including resistant pathogens such as *Listeria monocytogenes* [64,74]. It works by disrupting microbial membranes, interacting with membrane phospholipids, displacing or releasing enzymes, and ultimately leading to the lysis of the cell wall [75].

## 5. Beneficial Effects of Probiotic LABs

### 5.1. Improvements in Lactose Intolerance and Lactose Digestion

Lactose intolerance is a physiological disorder characterized by the inability of the human body to break down lactose due to a deficiency of the enzyme lactase in the intestinal mucosa [76]. This condition can result from digestive disorders or congenital diseases. Recent research indicates that probiotics can alleviate symptoms of lactose intolerance, which may include stomach pain, cramps, vomiting, and flatulence in adults, as well as acidic diarrhea in children [77]. Gingold-Belfer et al. [78] demonstrated that administering probiotics with β-galactosidase activity significantly improved symptoms in a vast majority of individuals with lactose malabsorption. Additionally, probiotics have been shown to effectively regulate the gut’s pH levels, enhance β-galactosidase expression, and modulate the colon’s microbiota [79].

### 5.2. Cholesterol-Lowering Activity

Some LAB probiotics have been shown to provide additional benefits, such as the absorption of cholesterol and the lowering of cholesterol and triglyceride levels in the blood. Hypercholesterolemia is recognized as a significant risk factor for the development of coronary heart disease [80]. Therefore, reducing blood cholesterol levels is crucial for disease prevention. *Lb. acidophilus* and *Enterococcus faecalis* isolated from traditional Chinese fermented cucumbers were found to have higher cholesterol- and triglyceride-lowering effects in vitro [81].

The exact mechanisms that cause the cholesterol-lowering effects of probiotics are not fully understood. According to Cao et al. [82], lactobacilli have the potential to modulate cholesterol levels by regulating the gene expression associated with cholesterol synthesis, metabolism, and absorption. Kazemian et al. [83] demonstrated that SCFAs can decrease serum lipid levels by inhibiting or redirecting cholesterol synthesis within the liver. In another study, Bosch et al. [84] indicated that three *Lpb. plantarum* strains (CECT 7527, 7528, and 7529) were capable of producing substantial quantities of propionic and butyric acids. These strains could also uptake cholesterol directly from the surrounding media and decrease its concentration through cell surface binding. Various alternative mechanisms have been proposed in order to account for the decline in intestinal cholesterol absorption following the regular consumption of probiotics. These mechanisms include the transformation of cholesterol into coprostanol, a sterol that cannot be absorbed by the body, and the inhibition of enterocyte expression of intestinal cholesterol transporters [85]. The EPSs derived from *Lactiplantibacillus* have also shown considerable potential in reducing cholesterol levels [86].

The research conducted by Modi et al. [87] demonstrated that a fermented amla beverage, using strains of *Pediococcus lolii*, *Pediococcus acidilactici*, *Pediococcus pentosaceus*, and *Lpb. plantarum*, positively impacts lipid profiles in both serum and the liver. These LAB strains modulate cholesterol levels through several mechanisms. They reduce cholesterol synthesis by lowering hepatic HMG-CoA reductase levels, counteract reactive oxygen species with their antioxidant properties to prevent cholesterol oxidation, and enhance liver function, which is critical for lipid metabolism. Additionally, they decrease serum triglycerides and total cholesterol. The beverage’s organic acids further inhibit cholesterol synthesis and improve fatty acid utilization, with these effects being enhanced by free polyphenols and flavonoids released by lactic acid fermentation.

### 5.3. Immunomodulation

Recent research has shown that the incorporation of probiotics into the diet enhances both the innate and adaptive immune responses [88]. Mazziotta et al. [3] have clarified how probiotics interact with the host’s immune system within the gut, detailing the mechanisms involved. These mechanisms include probiotics’ ability to interact with pattern recognition receptors (PRRs) on the surface of gut epithelial cells. This interaction triggers cytokine production and stimulates regulatory T cells (Tregs), which are crucial for maintaining immune homeostasis in the gut and limiting inflammation. Additionally, Tregs facilitate interactions with specialized enterocytes known as M cells. These cells assist in transporting antigens from the gut lumen to dendritic cells (DCs) in the lamina propria. The interaction between DCs and LABs leads to the activation of various T helper cell differentiation patterns or regulatory T cell responses [89]. Moreover, studies have demonstrated that LABs positively influence intestinal barrier function by enhancing mucus production from goblet cells. These cells form a physical barrier between the intestinal lumen and host tissues, preventing the invasion of harmful pathogens [90,91].

### 5.4. Modulation of Antioxidant Defense Systems and Anti-Inflammatory Properties

**Figure 2:**
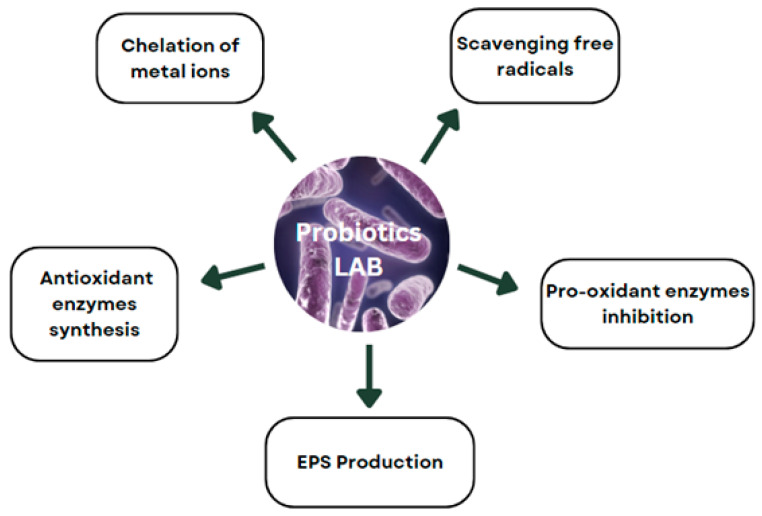
Diverse antioxidant mechanisms of LABs responsible for reducing oxidative stress.

The antioxidant and anti-inflammatory activities of LABs play a crucial role in managing oxidative stress and inflammatory processes in the body. Figure 2 illustrates the diverse antioxidant mechanisms of LABs responsible for reducing oxidative stress. LABs help neutralize reactive oxygen species (ROS), potentially harmful molecules that can damage cells and tissues. This neutralization primarily occurs through the chelation of metal ions that catalyze ROS formation via reactions such as the Fenton reaction [92]. These ROS are normal byproducts of cellular metabolism which, if unregulated, can damage cells and are associated with increased risks of heart disease, cancer, and arthritic conditions [93].

Moreover, certain strains of LABs activate or stimulate antioxidant enzymes, such as superoxide dismutase (SOD) and glutathione peroxidase (GPx), which break down ROS and protect cells from oxidative damage [94]. Wang et al. [95] discovered that when *Limosilactobacillus fermentum* (formerly known as *Lb. fermentum*) was added to the diets of pigs, it increased the total antioxidant capacity, particularly in larger pigs, due to enhanced levels of key antioxidative enzymes, including SOD and GPx. This was associated with a decrease in malondialdehyde levels, a biomarker of oxidative stress, suggesting reduced oxidative damage within the animals. A recent study by Yang et al. [96] demonstrated that *Lacticaseibacillus paracasei* (formerly known as *Lactobacillus paracasei*) M11-4 possesses inherent antioxidant activity and can upregulate its own antioxidant enzymes in response to hydrogen peroxide exposure.

In addition to reducing oxidative stress, LABs have significant anti-inflammatory properties. They modulate the inflammatory response by inhibiting the expression of key enzymes, such as cyclooxygenase-2 and inducible nitric oxide synthase, which are involved in the production of inflammatory mediators [97]. This inhibition helps to reduce the production of pro-inflammatory compounds, such as nitric oxide, thereby reducing inflammation and its associated effects.

### 5.5. Effects of LABs on Glycemic Control in Diabetes

The impact of LABs on blood sugar regulation is complex and multifaceted. A study of Ghafouri et al. [98] focusing on synbiotic bread containing lactic acid showed promising results, with a significant decrease in HbA1c levels in patients with type 2 diabetes, suggesting that LABs can positively influence metabolic health markers. On the other hand, a separate randomized controlled trial investigating the supplementation with *Lb. acidophilus* La5 and *Bifidobacterium animalis* subsp. *lactis* Bb12 revealed no substantial improvements in key diabetic biomarkers, such as fasting glucose, insulin, or HOMA-IR [99].

Recently, Jeong et al. [100] demonstrated that certain LAB strains exhibit noteworthy α-glucosidase inhibitory activities, which are instrumental in managing oxidative stress and moderating postprandial blood glucose levels, which are critical elements in diabetes management. This study also highlighted the anti-adipogenesis properties of these strains, establishing a link between obesity management and type 2 diabetes control.

### 5.6. Prevention of Intestinal Infections and Treatment of Diarrheal Diseases

The potential of probiotics to treat diarrheal diseases and prevent gastrointestinal infections has been well studied. Some probiotic strains help treat and prevent disease because they prevent bacteria from growing in the stomach. Clinical data indicate that various lactobacilli species appear to work efficiently in both viable and non-viable forms [21]. However, researchers recommend taking live probiotics because they can colonize and settle in the digestive tract, stabilize the gut flora, and eliminate pathogens [101]. According to a study by Guarino et al. [102], *Lacticaseibacillus rhamnosus* GG is helpful in both the prevention and treatment of diarrheal diseases. Probiotics have also been shown to stop antibiotic-induced diarrhea and reduce its severity, frequency, and duration as they can restore the balance of an imbalanced flora, improve water intake, minimize opportunistic infections, and strengthen the intestinal barrier and immunity [103].

Other gastrointestinal diseases such as irritable bowel syndrome and inflammatory bowel diseases (IBD) have also been successfully prevented by probiotics [104]. Environmental factors that cause dysbiosis are one of the main causes of IBD. Indeed, the gut microbiome of individuals with IBD exhibits a reduced abundance of bacterial species and genera in comparison to those who are considered to be in good health [105]. It has been shown that changes in the qualitative and quantitative composition of the gut microbiome are associated with chronic inflammation of the gut mucosa [106].

Probiotics may help people with IBD to reduce inflammation, improve symptoms, and delay recurrence, especially when used in conjunction with drug therapy [107]. A study conducted by Shadnoush et al. [108] has suggested that the consumption of probiotic yogurt containing *Bifidobacterium* and lactobacilli may be efficacious in reducing inflammation.

### 5.7. Cancer Prevention and Treatment

LAB probiotics have demonstrated significant potential in reducing the prevalence of cancer symptoms [15]. In particular, they have demonstrated antiproliferative or proapoptotic effects on a range of cancer cells, including colon, gastric, breast, cervical, and myeloid leukemia cells. Their potential to prevent and treat cancer may be due to a variety of factors (Table 1).

Probiotics may reduce cancer risk through several mechanisms [120]. They modulate the gut microbiota to maintain a balanced microbial environment and improve the integrity of the gut barrier, which prevents harmful substances from entering the bloodstream. Furthermore, they reduce DNA damage in the gut epithelium, potentially lowering the risk of cancer-causing mutations [121]. Probiotics also enhance the body’s immune and inflammatory responses, which helps fight infections and reduce the inflammation often linked with cancer progression [120]. A critical aspect of their anticancer effect involves the suppression of enzyme production in the gut microbiota that converts amines and aromatic substances into active carcinogens. This suppression can significantly decrease the formation of these carcinogens, thereby potentially preventing colorectal cancer [120]. Śliżewska et al. [120] highlight that inhibiting these enzymes is a promising strategy for cancer prevention. Additionally, Sharma et al. [122] have shown that metabiotics derived from *Lcb. rhamnosus* exhibit strong antigenotoxic and cytotoxic effects against colon cancer cells, such as Caco-2 and HT-29, illustrating the direct anticancer properties of probiotic derivatives. Regular intake of lactobacilli and *Bifidobacterium* for four to six weeks has also been associated with a lowered risk of colorectal cancer, further underscoring the protective potential of these probiotics [123].

Probiotics have also been known for their cytotoxic effects on various gastric and colon cancers by producing SCFAs (mainly acetate, propionate, and butyrate) [115]. These SCFAs not only energize colonocytes but also induce acidosis and cancer cell death, reducing secondary bile acid production. Among SCFAs, butyric acid is crucial in regulating colonocyte proliferation, division, and apoptosis, and it is present at higher levels in the feces of healthy individuals compared to those with colorectal cancer [124].

Many studies showed the potential use of EPSs from LABs as a therapeutic intervention in colorectal cancer. Deepak et al. [125] conducted a study that examined the impact of administering EPSs derived from the probiotic *Lb. acidophilus*. Their research focused on a rat model of 1,2-dimethylhydrazine-induced colon cancer, a well-established experimental setup for studying colorectal cancer. The results showed that the administration of EPSs is correlated with a decrease in the formation of polyps, which are precursors to colorectal cancer.

### 5.8. The Effect of LAB Probiotics on Mental Health and Cognitive Performance

The utilization of probiotics has been discovered to positively influence mental health and cognitive performance wellbeing (Table 2).

LAB probiotics influence cognitive performance through their interaction with the gut–brain axis, which is the bidirectional communication pathway between the gastrointestinal tract and the central nervous system [9]. LABs contribute to this interaction by producing various metabolites during fermentation, such as organic acids, amino acids, exopolysaccharides, and vitamins. These compounds help to modulate the gut microbiota, which is crucial for maintaining a balanced intestinal environment that supports overall health, including brain function. Additionally, some LAB strains can produce neurotransmitters, like gamma-aminobutyric acid (GABA), which is a key inhibitor in the mammalian central nervous system that plays a significant role in regulating mood and cognitive functions [129].

Moreover, LABs influence the immune system, which is intricately linked with the central nervous system [135]. By modulating immune responses and reducing inflammation, LABs can indirectly affect brain health and cognitive processes [136]. The direct impact of LABs on neural pathways further underscores their potential role in influencing cognitive functions [137]. Papalini et al. [138] demonstrated that a four-week probiotic treatment enhanced mental performance under stress, which is associated with changes in frontal lobe areas involved in cognitive regulation.

## 6. Next-Generation Probiotics and Postbiotics: Addressing Some of the Limitations of Probiotics

The probiotic products sector has gained popularity and global appeal in recent years. However, it is not always certain that the products sold deserve the claimed benefits, necessitating rigorous regulatory oversight. Zawistowska-Rojek et al. [139] found that many probiotic products on the market do not meet the claimed health benefits or the declared bacterial concentration, and some contain unlisted microorganisms, raising concerns about consumer safety and product transparency.

Another significant challenge in developing reliable and effective probiotic products lies in maintaining their functionality under various processing and storage conditions to fully realize their potential for enhancing human health. While lyophilization is commonly employed for microorganism preservation, it can lead to considerable viability loss for certain probiotic strains due to stress conditions, like extreme temperatures and dehydration. To address this issue, optimizing cryoprotectants becomes essential to enhance strain survival during lyophilization [140].

Moreover, microencapsulation presents another promising approach. This method entails encapsulating probiotics in microgels or lipids, offering substantial protection during gastrointestinal transit and improving product handling and shelf life. Afzaal et al. [141] have shown that encapsulated probiotics exhibit significantly higher survival rates than their non-encapsulated ones, particularly under simulated gastrointestinal conditions. They also demonstrate enhanced resistance within the intestinal tract and improved stability during storage, retaining their probiotic properties over time. However, it is worth noting that implementing these advanced technologies may lead to increased production costs.

Next-generation probiotics (NGPs) represent a significant evolution in the field of probiotics, defined as specifically selected strains of microorganisms that offer superior health benefits compared to traditional probiotics [142]. Developed using advanced techniques, such as next-generation sequencing, NGPs provide more precise targeting of health needs, improving the viability and effectiveness of probiotic strains under various processing and storage conditions. This innovation not only enhances the resistance and survival of probiotics in the gastrointestinal tract but also increases the potential for treating specific diseases through the production of targeted bioactive compounds [143]. However, NGPs have certain limitations. The safety of NGPs, not yet fully established due to their novelty, poses a major challenge. Their regulation, as biotherapeutic products, requires extensive clinical trials, which can delay their introduction into the food market.

To overcome these challenges, the utilization of postbiotics presents a significant opportunity. They are defined as preparations of inanimate microorganisms and/or their components that confer health benefits [144]. This approach addresses concerns associated with live cell administration, mitigating the risks for immunocompromised or vulnerable individuals. Opting for postbiotics brings numerous advantages [145], including the low likelihood of transfer of antimicrobial resistance genes to virulent microbes, which significantly reduces the incidence of infection [145]. However, for postbiotics to be considered safe, an ad hoc safety assessment cannot be omitted. This evaluation must encompass not only the parent microorganism but also the actual formulated amount and potential overdoses. Postbiotics derived from food-grade microorganisms or those listed on EFSA’s QPS lists may encounter smoother approval processes [146]. Postbiotics, unlike living cells, can be produced under controlled processes that ensure functional property maintenance during storage. They do not require special conditions to maintain viability, allowing for more stable and effective products [145]. Postbiotics derived from *Lpb. plantarum* have demonstrated cytotoxic effects and the ability to induce apoptosis in cancer cells, suggesting that they have potential to be used as supplements or adjunct treatments for cancer [147]. Promising results have also been obtained with heat-inactivated cells from *Lcb. paracasei*, *Lb. acidophilus*, and *Lcb. rhamnosus* [146].

Furthermore, postbiotics align with consumer preferences for natural ingredients, serving as clean-label alternatives. As techno-functional substances, they offer multiple benefits, enhancing food and beverage shelf life, sensory characteristics, and nutritional value without relying on chemical additives [148]. Regulatory authorities have not yet established a specific framework for foods or dietary supplements containing postbiotics. However, in certain countries, some postbiotic-based products are marketed under different regulatory categories. On the other hand, specific standards have been detailed for postbiotic formulations designated for pharmaceutical applications [146].

## 7. Conclusions

The functional attributes of LABs have received noteworthy considerations from the medical and food sectors due to their extensive health benefits. The integration of LAB-rich foods and probiotic dietary supplements into regular diets holds the potential for optimizing health-enhancing outcomes. Nevertheless, it is critical to conduct further research to clarify the precise mechanisms for the development of LAB-based targeted therapeutic approaches. Furthermore, exploring alternative approaches, such as postbiotics, offers a promising path to enhance therapeutic outcomes and promote human wellbeing. Postbiotics offer various benefits in comparison to live cells, encompassing amplified safety and stability as well as a wider range of potential applications. Furthermore, postbiotics can act as techno-functional additives in the food industry, contributing not only to product advancement but also aligning with the developing consumer demands for natural, health-promoting ingredients. The continuous research and application of postbiotics in these sectors can lead to the development of more salubrious, secure, and functional food products. Additionally, the NGPs can amplify the potential for targeted health benefits, utilizing specially engineered strains that enhance both efficacy and specificity. Novel, effective biotechnological tools can be developed by harnessing whole microbial consortia or by the de novo assembly of synthetic microbial communities, further expanding the scope of beneficial microbial applications in health and nutrition.

The monoculture approach has long overlooked the role that ecological interactions play in the functioning and growth of LABs within microbial consortia [149]. These consortia underlie food chains and provide essential support for ecosystems and macroscopic organisms. Microbial consortia consist of both culturable and unculturable isolates with significant physiological and biochemical differences. Although modern culture-independent approaches have allowed tremendous advances in our knowledge of the non-culturable portions of microbial consortia, their techno-functional exploitation remains a challenge. There is immense biotechnological potential in the ability to harness entire microbial consortia rather than mono-cultures or simple co-cultures. Microbial consortia offer greater resilience, complex functionalities, and a reduced metabolic burden with respect to single-species cultures [150]. While the benefits of harnessing the full functional potential of whole microbial consortia are well understood, there are conceptual and technical hurdles in the way of achieving this goal. Overcoming such obstacles requires the ability to propagate and maintain entire natural microbial consortia. Hence, it is imperative to optimize targeted preservation methodologies to uphold the structure and metabolic functions of microbial consortia, ensuring the integrity and characteristics of the associated ecosystem. Genetic alterations and viability decline are particularly significant in microbial consortia compared to pure cultures, due to different tolerance levels of microbial components (species and strains) to storage-induced stresses. Examples of efforts in this direction are the technique of human fecal microbiota transplantation [151] or the assembly of synthetic sourdough metacommunities [147]. Taking advantage of microbial consortia, synthetic metacommunities comprising predetermined compositions can be assembled for a variety of biotechnological purposes. The ability to predict and assemble large multispecies communities requires an understanding of how these microbiomes form, function, and coexist, ultimately enabling the design of functional microbiomes de novo. Predicting the composition and behavior of microbial communities cannot rely solely on aggregating individual components or monitoring the most abundant species. Ad hoc approaches are needed to anticipate metacommunity dynamics and drive coexisting species toward resilient states [147,152].

## Figures and Tables

**Table 1:** Potential roles of LABs in cancer prevention and treatment.

Action Mode	Description	References
Modulation of gut microbiome	LABs modulate the gut microbiome to reduce inflammation and oxidative stress. This includes altering metabolic activities of the intestinal microflora, binding/degrading potential carcinogens, and producing antitumorigenic compounds.	[109,110,111]
Boosting the immune response	Activation of natural killer cells and cytotoxic T lymphocytes. LABs can increase cytokines, such as TNF-α, IFN-γ, and IL-10, enhancing cellular immunity and suppressing tumor cell proliferation.	[111,112]
Production of anticancer metabolites	LABs produce anticancer metabolites, such as extracellular polysaccharides, peptidoglycans, bacteriocins, and SCFAs, inhibiting cancer cell growth and inducing apoptosis. LAB-produced EPSs can suppress tumor-related enzymes, while SCFAs maintain gut health and reduce inflammation.	[113,114,115]
Gut–brain axis modulation	The complex interaction between gut microbiota and the brain, influencing stress and inflammation, is critical in cancer development. Dysbiosis may lead to a ‘leaky gut’, elevating inflammation and cancer risk. The gut microbiota’s role in neurotransmitter production and immune system modulation is also significant.	[116]
Elevating quality of life for cancer patients	Alleviating fatigue, enhancing mood, and improving the quality of life for cancer patients overall.	[117,118]
Synergistic effects with chemotherapy/radiotherapy	Observed synergistic effects when combined with chemotherapy or radiotherapy.	[116,119]

**Table 2:** The role of probiotics on mental health and wellbeing.

Mental Health and Wellbeing	Role of Probiotics	References
Alleviating symptoms of anxiety and depression	Modulates the hypothalamic–pituitary–adrenal axis, the primary stress response system in the body. Fine tunes the gut–brain axis to optimize communication between the gut and the brain. Generates neuroactive metabolites that influence brain function and behavior.	[126,127,128,129]
Promoting reduction in the incidence of neurodegenerative diseases and enhancing cognitive performance	Reduces oxidative stress and inflammatory cytokines, targeting Alzheimer’s disease biomarkers. Inhibits apoptosis and boosts neurotrophic factors, like BDNF, which are crucial for neuron survival. Protects dopaminergic neurons and suppresses neuroinflammation, enhancing antioxidant capacity. Produces SCFAs, which are associated with improved cognitive function.	[130,131,132,133,134]

## Data Availability

The original contributions presented in the study are included in the article, further inquiries can be directed to the corresponding author.

## Abbreviations

BDNF	Brain-Derived Neurotrophic Factor
CFU	Colony-forming unit
CLA	Conjugated linoleic acid
DPPH	2,2-Diphenyl-1-picrylhydrazyl
EPSs	Exopolysaccharides
FAO/WHO	Food and Agriculture Organization/World Health Organization
GABA	Gamma-aminobutyric acid
GPx	Glutathione peroxidase
GRAS	Generally Recognized as Safe
HICA	2-hydroxyisocaproic acid
HOMA-IR	Homeostatic Model Assessment for Insulin Resistance
IBD	Inflammatory bowel disease
LABs	Lactic acid bacteria
*Lb.*	*Lactobacillus*
*Lpb.*	*Lactiplantibacillus*
*Lcb.*	*Lacticaseibacillus*
*Ln.*	*Leuconostoc*
MnSOD	Manganese superoxide dismutase
ROS	Reactive oxygen species
SCFAs	Short-chain fatty acids
SOD	Superoxide dismutase

## References

[1] Abdul Hakim B.N., Xuan N.J., Oslan S.N.H. (2023). A Comprehensive Review of Bioactive Compounds from Lactic Acid Bacteria: Potential Functions as Functional Food in Dietetics and the Food Industry. Foods.

[2] Ibrahim S.A., Ayivi R.D., Zimmerman T., Siddiqui S.A., Altemimi A.B., Fidan H., Esatbeyoglu T., Bakhshayesh R.V. (2021). Lactic Acid Bacteria as Antimicrobial Agents: Food Safety and Microbial Food Spoilage Prevention. Foods.

[3] Mazziotta C., Tognon M., Martini F., Torreggiani E., Rotondo J.C. (2023). Probiotics Mechanism of Action on Immune Cells and Beneficial Effects on Human Health. Cells.

[4] Yoo J.Y., Groer M., Dutra S.V.O., Sarkar A., McSkimming D.I. (2020). Gut Microbiota and Immune System Interactions. Microorganisms.

[5] Madison A., Kiecolt-Glaser J.K. (2019). Stress, Depression, Diet, and the Gut Microbiota: Human–Bacteria Interactions at the Core of Psychoneuroimmunology and Nutrition. Curr. Opin. Behav. Sci..

[6] Martinelli M., Banderali G., Bobbio M., Civardi E., Chiara A., D’Elios S., Lo Vecchio A., Olivero M., Peroni D., Romano C. (2020). Probiotics’ Efficacy in Paediatric Diseases: Which Is the Evidence? A Critical Review on Behalf of the Italian Society of Pediatrics. Ital. J. Pediatr..

[7] Cheng D., Song J., Xie M., Song D. (2019). The Bidirectional Relationship between Host Physiology and Microbiota and Health Benefits of Probiotics: A Review. Trends Food Sci. Technol..

[8] Zheng J., Wittouck S., Salvetti E., Franz C.M.A.P., Harris H.M.B., Mattarelli P., O’Toole P.W., Pot B., Vandamme P., Walter J. (2020). A Taxonomic Note on the Genus *Lactobacillus*: Description of 23 Novel Genera, Emended Description of the Genus *Lactobacillus* Beijerinck 1901, and Union of *Lactobacillaceae* and *Leuconostocaceae*. Int. J. Syst. Evol. Microbiol..

[9] Tang H., Huang W., Yao Y.-F. (2023). The Metabolites of Lactic Acid Bacteria: Classification, Biosynthesis and Modulation of Gut Microbiota. Microb. Cell.

[10] Erten H., Ağirman B., Gündüz C.P.B., Çarşanba E., Sert S., Bircan S., Tangüler H., Malik A., Erginkaya Z., Ahmad S., Erten H. (2014). Importance of Yeasts and Lactic Acid Bacteria in Food Processing. Food Processing: Strategies for Quality Assessment.

[11] Szutowska J. (2020). Functional Properties of Lactic Acid Bacteria in Fermented Fruit and Vegetable Juices: A Systematic Literature Review. Eur. Food Res. Technol..

[12] Hill C., Guarner F., Reid G., Gibson G.R., Merenstein D.J., Pot B., Morelli L., Canani R.B., Flint H.J., Salminen S. (2014). The International Scientific Association for Probiotics and Prebiotics Consensus Statement on the Scope and Appropriate Use of the Term Probiotic. Nat. Rev. Gastroenterol. Hepatol..

[13] Ayivi R.D., Gyawali R., Krastanov A., Aljaloud S.O., Worku M., Tahergorabi R., da Silva R.C., Ibrahim S.A. (2020). Lactic Acid Bacteria: Food Safety and Human Health Applications. Dairy.

[14] Binda S., Hill C., Johansen E., Obis D., Pot B., Sanders M.E., Tremblay A., Ouwehand A.C. (2020). Criteria to Qualify Microorganisms as “Probiotic” in Foods and Dietary Supplements. Front. Microbiol..

[15] Nazir Y., Hussain S.A., Abdul Hamid A., Song Y. (2018). Probiotics and Their Potential Preventive and Therapeutic Role for Cancer, High Serum Cholesterol, and Allergic and HIV Diseases. BioMed Res. Int..

[16] Harper A.R., Dobson R.C.J., Morris V.K., Moggré G.-J. (2022). Fermentation of Plant-Based Dairy Alternatives by Lactic Acid Bacteria. Microb. Biotechnol..

[17] Grasso N., Alonso-Miravalles L., O’Mahony J.A. (2020). Composition, Physicochemical and Sensorial Properties of Commercial Plant-Based Yogurts. Foods.

[18] Hickey C.D., Sheehan J.J., Wilkinson M.G., Auty M.A.E. (2015). Growth and Location of Bacterial Colonies within Dairy Foods Using Microscopy Techniques: A Review. Front. Microbiol..

[19] Warner R. (2017). The Eating Quality of Meat—IV Water-Holding Capacity and Juiciness. Lawrie’s Meat Science.

[20] Barbut S. (2005). Effects of Chemical Acidification and Microbial Fermentation on the Rheological Properties of Meat Products. Meat Sci..

[21] Cao C.-C., Feng M.-Q., Sun J., Xu X.-L., Zhou G.-H. (2019). Screening of Lactic Acid Bacteria with High Protease Activity from Fermented Sausages and Antioxidant Activity Assessment of Its Fermented Sausages. CyTA—J. Food.

[22] Chen W., Xie C., He Q., Sun J., Bai W. (2023). Improvement in Color Expression and Antioxidant Activity of Strawberry Juice Fermented with Lactic Acid Bacteria: A Phenolic-Based Research. Food Chem. X.

[23] Ayed L., M’hir S., Hamdi M. (2020). Microbiological, Biochemical, and Functional Aspects of Fermented Vegetable and Fruit Beverages. J. Chem..

[24] Valero-Cases E., Nuncio-Jáuregui N., Frutos M.J. Influence of Fermentation with Different Lactic Acid Bacteria and In Vitro Digestion on the Biotransformation of Phenolic Compounds in Fermented Pomegranate Juices. https://pubs.acs.org/doi/pdf/10.1021/acs.jafc.6b04854.

[25] Vicente J., Baran Y., Navascués E., Santos A., Calderón F., Marquina D., Rauhut D., Benito S. (2022). Biological Management of Acidity in Wine Industry: A Review. Int. J. Food Microbiol..

[26] Pérez-Alvarado O., Zepeda-Hernández A., Garcia-Amezquita L.E., Requena T., Vinderola G., García-Cayuela T. (2022). Role of Lactic Acid Bacteria and Yeasts in Sourdough Fermentation during Breadmaking: Evaluation of Postbiotic-like Components and Health Benefits. Front. Microbiol..

[27] Maryati Y., Nuraida L., Hariyadi R.D. (2021). Production of Organic Acid and Short-Chain Fatty Acids (SCFA) from Lactic Acid Bacteria Isolate on Oligosaccharide Media. J. Kim. Sains Apl..

[28] Lee S.-J., Jeon H.-S., Yoo J.-Y., Kim J.-H. (2021). Some Important Metabolites Produced by Lactic Acid Bacteria Originated from Kimchi. Foods.

[29] Kieliszek M., Pobiega K., Piwowarek K., Kot A.M. (2021). Characteristics of the Proteolytic Enzymes Produced by Lactic Acid Bacteria. Molecules.

[30] Shihata A., Shah N.P. (2000). Proteolytic Profiles of Yogurt and Probiotic Bacteria. Int. Dairy J..

[31] García-Cano I., Rocha-Mendoza D., Kosmerl E., Zhang L., Jiménez-Flores R. (2020). Technically Relevant Enzymes and Proteins Produced by LAB Suitable for Industrial and Biological Activity. Appl. Microbiol. Biotechnol..

[32] Zheng X., Shi X., Wang B. (2021). A Review on the General Cheese Processing Technology, Flavor Biochemical Pathways and the Influence of Yeasts in Cheese. Front. Microbiol..

[33] Geiselhart S., Podzhilkova A., Hoffmann-Sommergruber K. (2021). Cow’s Milk Processing-Friend or Foe in Food Allergy?. Foods.

[34] Abril B., Bou R., García-Pérez J.V., Benedito J. (2023). Role of Enzymatic Reactions in Meat Processing and Use of Emerging Technologies for Process Intensification. Foods.

[35] Emkani M., Oliete B., Saurel R. (2022). Effect of Lactic Acid Fermentation on Legume Protein Properties, a Review. Fermentation.

[36] De Vuyst L., Van Kerrebroeck S., Leroy F. (2017). Microbial Ecology and Process Technology of Sourdough Fermentation. Adv. Appl. Microbiol..

[37] Jurášková D., Ribeiro S.C., Silva C.C.G. (2022). Exopolysaccharides Produced by Lactic Acid Bacteria: From Biosynthesis to Health-Promoting Properties. Foods.

[38] Abarquero D., Renes E., Fresno J.M., Tornadijo M.E. (2022). Study of Exopolysaccharides from Lactic Acid Bacteria and Their Industrial Applications: A Review. Int. J. Food Sci. Technol..

[39] Daba G.M., Elnahas M.O., Elkhateeb W.A. (2021). Contributions of Exopolysaccharides from Lactic Acid Bacteria as Biotechnological Tools in Food, Pharmaceutical, and Medical Applications. Int. J. Biol. Macromol..

[40] London L.E.E., Chaurin V., Auty M.A.E., Fenelon M.A., Fitzgerald G.F., Ross R.P., Stanton C. (2015). Use of *Lactobacillus mucosae* DPC 6426, an Exopolysaccharide-Producing Strain, Positively Influences the Techno-Functional Properties of Yoghurt. Int. Dairy J..

[41] Galli V., Venturi M., Coda R., Maina N.H., Granchi L. (2020). Isolation and Characterization of Indigenous *Weissella confusa* for in Situ Bacterial Exopolysaccharides (EPS) Production in Chickpea Sourdough. Food Res. Int..

[42] Thierry A., Pogačić T., Weber M., Lortal S. (2015). Production of Flavor Compounds by Lactic Acid Bacteria in Fermented Foods. Biotechnology of Lactic Acid Bacteria.

[43] Pétel C., Onno B., Prost C. (2017). Sourdough Volatile Compounds and Their Contribution to Bread: A Review. Trends Food Sci. Technol..

[44] Wang Y., Wu J., Lv M., Shao Z., Hungwe M., Wang J., Bai X., Xie J., Wang Y., Geng W. (2021). Metabolism Characteristics of Lactic Acid Bacteria and the Expanding Applications in Food Industry. Front. Bioeng. Biotechnol..

[45] Laëtitia G., Pascal D., Yann D. (2014). The Citrate Metabolism in Homo- and Heterofermentative LAB: A Selective Means of Becoming Dominant over Other Microorganisms in Complex Ecosystems. Food Nutr. Sci..

[46] García-Cano I., Rocha-Mendoza D., Ortega-Anaya J., Wang K., Kosmerl E., Jiménez-Flores R. (2019). Lactic Acid Bacteria Isolated from Dairy Products as Potential Producers of Lipolytic, Proteolytic and Antibacterial Proteins. Appl. Microbiol. Biotechnol..

[47] Wang Y., Han J., Wang D., Gao F., Zhang K., Tian J., Jin Y. (2022). Research Update on the Impact of Lactic Acid Bacteria on the Substance Metabolism, Flavor, and Quality Characteristics of Fermented Meat Products. Foods.

[48] Guan C., Tao Z., Wang L., Zhao R., Chen X., Huang X., Su J., Lu Z., Chen X., Gu R. (2020). Isolation of Novel *Lactobacillus* with Lipolytic Activity from the Vinasse and Their Preliminary Potential Using as Probiotics. AMB Express.

[49] Picon A., López-Pérez O., Torres E., Garde S., Nuñez M. (2019). Contribution of Autochthonous Lactic Acid Bacteria to the Typical Flavour of Raw Goat Milk Cheeses. Int. J. Food Microbiol..

[50] Ardö Y. (2006). Flavour Formation by Amino Acid Catabolism. Biotechnol. Adv..

[51] Liu S., Sun H., Ma G., Zhang T., Wang L., Pei H., Li X., Gao L. (2022). Insights into Flavor and Key Influencing Factors of Maillard Reaction Products: A Recent Update. Front. Nutr..

[52] Papaioannou G., Kosma I., Badeka A.V., Kontominas M.G. (2021). Profile of Volatile Compounds in Dessert Yogurts Prepared from Cow and Goat Milk, Using Different Starter Cultures and Probiotics. Foods.

[53] Fadlillah H.N., Nuraida L., Sitanggang A.B., Palupi N.S. (2021). Production of Antioxidants through Lactic Acid Fermentation: Current Developments and Outlook. Ann. Univ. Dunarea Jos Galati Fascicle VI—Food Technol..

[54] Ayed L., Hamdi M. (2002). Culture Condition of Tannase Production by *Lactobacillus plantarum*. Biotechnol. Lett..

[55] Filannino P., Gobbetti M., De Angelis M., Di Cagno R. (2014). Hydroxycinnamic Acids Used as External Acceptors of Electrons: An Energetic Advantage for Strictly Heterofermentative Lactic Acid Bacteria. Appl. Environ. Microbiol..

[56] Fardet A., Rock E. (2018). In Vitro and in Vivo Antioxidant Potential of Milks, Yoghurts, Fermented Milks and Cheeses: A Narrative Review of Evidence. Nutr. Res. Rev..

[57] Tadesse S.A., Emire S.A. (2020). Production and Processing of Antioxidant Bioactive Peptides: A Driving Force for the Functional Food Market. Heliyon.

[58] Verni M., Verardo V., Rizzello C.G. (2019). How Fermentation Affects the Antioxidant Properties of Cereals and Legumes. Foods.

[59] Wang X., Shao C., Liu L., Guo X., Xu Y., Lü X. (2017). Optimization, Partial Characterization and Antioxidant Activity of an Exopolysaccharide from *Lactobacillus plantarum* KX041. Int. J. Biol. Macromol..

[60] Kalhoro M.S., Anal A.K., Kalhoro D.H., Hussain T., Murtaza G., Mangi M.H. (2023). Antimicrobial Activities and Biopreservation Potential of Lactic Acid Bacteria (LAB) from Raw Buffalo (Bubalus Bubalis) Milk. Oxid. Med. Cell. Longev..

[61] Abouloifa H., Hasnaoui I., Rokni Y., Bellaouchi R., Ghabbour N., Karboune S., Brasca M., Abousalham A., Jaouadi B., Saalaoui E. (2022). Antifungal Activity of Lactic Acid Bacteria and Their Application in Food Biopreservation. Adv. Appl. Microbiol..

[62] Guimarães A., Venancio A., Abrunhosa L. (2018). Antifungal Effect of Organic Acids from Lactic Acid Bacteria on Penicillium Nordicum. Food Addit. Contam. Part A.

[63] Gómez-García M., Sol C., de Nova P.J.G., Puyalto M., Mesas L., Puente H., Mencía-Ares Ó., Miranda R., Argüello H., Rubio P. (2019). Antimicrobial Activity of a Selection of Organic Acids, Their Salts and Essential Oils against Swine Enteropathogenic Bacteria. Porc. Health Manag..

[64] Cirat R., Capozzi V., Benmechernene Z., Spano G., Grieco F., Fragasso M. (2024). LAB Antagonistic Activities and Their Significance in Food Biotechnology: Molecular Mechanisms, Food Targets, and Other Related Traits of Interest. Fermentation.

[65] Shi C., Maktabdar M. (2022). Lactic Acid Bacteria as Biopreservation against Spoilage Molds in Dairy Products—A Review. Front. Microbiol..

[66] Peng M., Biswas D. (2017). Short Chain and Polyunsaturated Fatty Acids in Host Gut Health and Foodborne Bacterial Pathogen Inhibition. Crit. Rev. Food Sci. Nutr..

[67] Werning M.L., Hernández-Alcántara A.M., Ruiz M.J., Soto L.P., Dueñas M.T., López P., Frizzo L.S. (2022). Biological Functions of Exopolysaccharides from Lactic Acid Bacteria and Their Potential Benefits for Humans and Farmed Animals. Foods.

[68] Wang J., Zhao X., Yang Y., Zhao A., Yang Z. (2015). Characterization and Bioactivities of an Exopolysaccharide Produced by *Lactobacillus plantarum* YW32. Int. J. Biol. Macromol..

[69] Abdalla A.K., Ayyash M.M., Olaimat A.N., Osaili T.M., Al-Nabulsi A.A., Shah N.P., Holley R. (2021). Exopolysaccharides as Antimicrobial Agents: Mechanism and Spectrum of Activity. Front. Microbiol..

[70] Nehal F., Sahnoun M., Smaoui S., Jaouadi B., Bejar S., Mohammed S. (2019). Characterization, High Production and Antimicrobial Activity of Exopolysaccharides from *Lactococcus lactis* F-mou. Microb. Pathog..

[71] Simons A., Alhanout K., Duval R.E. (2020). Bacteriocins, Antimicrobial Peptides from Bacterial Origin: Overview of Their Biology and Their Impact against Multidrug-Resistant Bacteria. Microorganisms.

[72] Dicks L.M.T., Dreyer L., Smith C., van Staden A.D. (2018). A Review: The Fate of Bacteriocins in the Human Gastro-Intestinal Tract: Do They Cross the Gut–Blood Barrier?. Front. Microbiol..

[73] Müller-Auffermann K., Grijalva F., Jacob F., Hutzler M. (2015). Nisin and Its Usage in Breweries: A Review and Discussion. J. Inst. Brew..

[74] Shin J.M., Gwak J.W., Kamarajan P., Fenno J.C., Rickard A.H., Kapila Y.L. (2016). Biomedical Applications of Nisin. J. Appl. Microbiol..

[75] Paiva A.D., Breukink E., Mantovani H.C. (2011). Role of Lipid II and Membrane Thickness in the Mechanism of Action of the Lantibiotic Bovicin HC5. Antimicrob. Agents Chemother..

[76] Misselwitz B., Butter M., Verbeke K., Fox M.R. (2019). Update on Lactose Malabsorption and Intolerance: Pathogenesis, Diagnosis and Clinical Management. Gut.

[77] Malik T.F., Panuganti K.K. (2023). Lactose Intolerance. StatPearls.

[78] Gingold-Belfer R., Levy S., Layfer O., Pakanaev L., Niv Y., Dickman R., Perets T.T. (2020). Use of a Novel Probiotic Formulation to Alleviate Lactose Intolerance Symptoms-a Pilot Study. Probiotics Antimicrob. Proteins.

[79] Ibrahim S.A., Gyawali R., Awaisheh S.S., Ayivi R.D., Silva R.C., Subedi K., Aljaloud S.O., Siddiqui S.A., Krastanov A. (2021). Fermented Foods and Probiotics: An Approach to Lactose Intolerance. J. Dairy Res..

[80] Hajar R. (2017). Risk Factors for Coronary Artery Disease: Historical Perspectives. Heart Views Off. J. Gulf Heart Assoc..

[81] Gao Y., Li D. (2018). Screening of Lactic Acid Bacteria with Cholesterol-Lowering and Triglyceride-Lowering Activity in Vitro and Evaluation of Probiotic Function. Ann. Microbiol..

[82] Cao K., Zhang K., Ma M., Ma J., Tian J., Jin Y. (2021). *Lactobacillus* Mediates the Expression of NPC1L1, CYP7A1, and ABCG5 Genes to Regulate Cholesterol. Food Sci. Nutr..

[83] Kazemian N., Mahmoudi M., Halperin F., Wu J.C., Pakpour S. (2020). Gut Microbiota and Cardiovascular Disease: Opportunities and Challenges. Microbiome.

[84] Bosch M., Fuentes M.C., Audivert S., Bonachera M.A., Peiró S., Cuñé J. (2014). *Lactobacillus plantarum* CECT 7527, 7528 and 7529: Probiotic Candidates to Reduce Cholesterol Levels. J. Sci. Food Agric..

[85] Reis S.A., Conceição L.L., Rosa D.D., Siqueira N.P., Peluzio M.C.G. (2017). Mechanisms Responsible for the Hypocholesterolaemic Effect of Regular Consumption of Probiotics. Nutr. Res. Rev..

[86] Sasikumar K., Kozhummal Vaikkath D., Devendra L., Nampoothiri K.M. (2017). An Exopolysaccharide (EPS) from a *Lactobacillus plantarum* BR2 with Potential Benefits for Making Functional Foods. Bioresour. Technol..

[87] Modi R., Sahota P., Singh N.D., Garg M. (2023). Hepatoprotective and Hypoglycemic Effect of Lactic Acid Fermented Indian Gooseberry-Amla Beverage on Chronic Alcohol-Induced Liver Damage and Diabetes in Rats. Food Hydrocoll. Health.

[88] Javanshir N., Hosseini G.N.G., Sadeghi M., Esmaeili R., Satarikia F., Ahmadian G., Allahyari N. (2021). Evaluation of the Function of Probiotics, Emphasizing the Role of Their Binding to the Intestinal Epithelium in the Stability and Their Effects on the Immune System. Biol. Proced. Online.

[89] Hardy H., Harris J., Lyon E., Beal J., Foey A.D. (2013). Probiotics, Prebiotics and Immunomodulation of Gut Mucosal Defences: Homeostasis and Immunopathology. Nutrients.

[90] Maldonado Galdeano C., Cazorla S.I., Lemme Dumit J.M., Vélez E., Perdigón G. (2019). Beneficial Effects of Probiotic Consumption on the Immune System. Ann. Nutr. Metab..

[91] Ma J., Piao X., Mahfuz S., Long S., Wang J. (2021). The Interaction among Gut Microbes, the Intestinal Barrier and Short Chain Fatty Acids. Anim. Nutr..

[92] Amaretti A., di Nunzio M., Pompei A., Raimondi S., Rossi M., Bordoni A. (2013). Antioxidant Properties of Potentially Probiotic Bacteria: In Vitro and in Vivo Activities. Appl. Microbiol. Biotechnol..

[93] Phaniendra A., Jestadi D.B., Periyasamy L. (2015). Free Radicals: Properties, Sources, Targets, and Their Implication in Various Diseases. Indian J. Clin. Biochem..

[94] Feng T., Wang J. (2020). Oxidative Stress Tolerance and Antioxidant Capacity of Lactic Acid Bacteria as Probiotic: A Systematic Review. Gut Microbes.

[95] Wang A.N., Yi X.W., Yu H.F., Dong B., Qiao S.Y. (2009). Free Radical Scavenging Activity of *Lactobacillus fermentum* in Vitro and Its Antioxidative Effect on Growing–Finishing Pigs. J. Appl. Microbiol..

[96] Yang J., Dong C., Ren F., Xie Y., Liu H., Zhang H., Jin J. (2022). *Lactobacillus paracasei* M11-4 Isolated from Fermented Rice Demonstrates Good Antioxidant Properties in Vitro and in Vivo. J. Sci. Food Agric..

[97] Kim S., Lee J.Y., Jeong Y., Kang C.-H. (2022). Antioxidant Activity and Probiotic Properties of Lactic Acid Bacteria. Fermentation.

[98] Ghafouri A., Zarrati M., Shidfar F., Heydari I., Shokouhi Shoormasti R., Eslami O. (2019). Effect of Synbiotic Bread Containing Lactic Acid on Glycemic Indicators, Biomarkers of Antioxidant Status and Inflammation in Patients with Type 2 Diabetes: A Randomized Controlled Trial. Diabetol. Metab. Syndr..

[99] Ivey K.L., Hodgson J.M., Kerr D.A., Lewis J.R., Thompson P.L., Prince R.L. (2014). The Effects of Probiotic Bacteria on Glycaemic Control in Overweight Men and Women: A Randomised Controlled Trial. Eur. J. Clin. Nutr..

[100] Jeong Y., Kim H., Lee J.Y., Won G., Choi S.-I., Kim G.-H., Kang C.-H. (2021). The Antioxidant, Anti-Diabetic, and Anti-Adipogenesis Potential and Probiotic Properties of Lactic Acid Bacteria Isolated from Human and Fermented Foods. Fermentation.

[101] Milner E., Stevens B., An M., Lam V., Ainsworth M., Dihle P., Stearns J., Dombrowski A., Rego D., Segars K. (2021). Utilizing Probiotics for the Prevention and Treatment of Gastrointestinal Diseases. Front. Microbiol..

[102] Guarino A., Guandalini S., Lo Vecchio A. (2015). Probiotics for Prevention and Treatment of Diarrhea. J. Clin. Gastroenterol..

[103] Kopacz K., Phadtare S. (2022). Probiotics for the Prevention of Antibiotic-Associated Diarrhea. Healthcare.

[104] Jonkers D., Stockbrügger R. (2003). Probiotics and Inflammatory Bowel Disease. J. R. Soc. Med..

[105] McIlroy J., Ianiro G., Mukhopadhya I., Hansen R., Hold G.L. (2018). Review Article: The Gut Microbiome in Inflammatory Bowel Disease—Avenues for Microbial Management. Aliment. Pharmacol. Ther..

[106] Chakaroun R.M., Massier L., Kovacs P. (2020). Gut Microbiome, Intestinal Permeability, and Tissue Bacteria in Metabolic Disease: Perpetrators or Bystanders?. Nutrients.

[107] Akutko K., Stawarski A. (2021). Probiotics, Prebiotics and Synbiotics in Inflammatory Bowel Diseases. J. Clin. Med..

[108] Shadnoush M., Shaker Hosseini R., Mehrabi Y., Delpisheh A., Alipoor E., Faghfoori Z., Mohammadpour N., Zaringhalam Moghadam J. (2013). Probiotic Yogurt Affects Pro- and Anti-Inflammatory Factors in Patients with Inflammatory Bowel Disease. Iran. J. Pharm. Res. IJPR.

[109] Chen Y., Yang B., Ross R.P., Jin Y., Stanton C., Zhao J., Zhang H., Chen W. (2019). Orally Administered CLA Ameliorates DSS-Induced Colitis in Mice via Intestinal Barrier Improvement, Oxidative Stress Reduction, and Inflammatory Cytokine and Gut Microbiota Modulation. J. Agric. Food Chem..

[110] Yang X., Yu D., Xue L., Li H., Du J. (2020). Probiotics Modulate the Microbiota–Gut–Brain Axis and Improve Memory Deficits in Aged SAMP8 Mice. Acta Pharm. Sin. B.

[111] Amenu D. (2017). Overview of Anticancer Activity of Lactic Acid Bacteria. Int. J. Adv. Res. Biol. Sci. IJARBS.

[112] Aziz N., Bonavida B. (2016). Activation of Natural Killer Cells by Probiotics. Forum Immunopathol. Dis. Ther..

[113] Dehghani N., Tafvizi F., Jafari P. (2021). Cell Cycle Arrest and Anti-Cancer Potential of Probiotic *Lactobacillus rhamnosus* against HT-29 Cancer Cells. BioImpacts BI.

[114] Wu J., Zhang Y., Ye L., Wang C. (2021). The Anti-Cancer Effects and Mechanisms of Lactic Acid Bacteria Exopolysaccharides in Vitro: A Review. Carbohydr. Polym..

[115] Markowiak-Kopeć P., Śliżewska K. (2020). The Effect of Probiotics on the Production of Short-Chain Fatty Acids by Human Intestinal Microbiome. Nutrients.

[116] Moughnyeh M.M., Brawner K.M., Kennedy B.A., Yeramilli V.A., Udayakumar N., Graham J.A., Martin C.A. (2021). Stress and the Gut-Brain Axis: Implications for Cancer, Inflammation and Sepsis. J. Surg. Res..

[117] Lee J.-Y., Chu S.-H., Jeon J.Y., Lee M.-K., Park J.-H., Lee D.-C., Lee J.-W., Kim N.-K. (2014). Effects of 12 Weeks of Probiotic Supplementation on Quality of Life in Colorectal Cancer Survivors: A Double-Blind, Randomized, Placebo-Controlled Trial. Dig. Liver Dis..

[118] Obermoser K., Brigo N., Schroll A., Monfort-Lanzas P., Gostner J.M., Engl S., Geisler S., Knoll M., Schennach H., Weiss G. (2023). Positive Effects of Probiotic Therapy in Patients with Post-Infectious Fatigue. Metabolites.

[119] Stage M., Wichmann A., Jørgensen M., Vera-Jimenéz N.I., Wielje M., Nielsen D.S., Sandelin A., Chen Y., Baker A. (2020). *Lactobacillus rhamnosus* GG Genomic and Phenotypic Stability in an Industrial Production Process. Appl. Environ. Microbiol..

[120] Śliżewska K., Markowiak-Kopeć P., Śliżewska W. (2020). The Role of Probiotics in Cancer Prevention. Cancers.

[121] Latif A., Shehzad A., Niazi S., Zahid A., Ashraf W., Iqbal M.W., Rehman A., Riaz T., Aadil R.M., Khan I.M. (2023). Probiotics: Mechanism of Action, Health Benefits and Their Application in Food Industries. Front. Microbiol..

[122] Sharma M., Chandel D., Shukla G. (2020). Antigenotoxicity and Cytotoxic Potentials of Metabiotics Extracted from Isolated Probiotic, *Lactobacillus rhamnosus* MD 14 on Caco-2 and HT-29 Human Colon Cancer Cells. Nutr. Cancer.

[123] Molska M., Reguła J. (2019). Potential Mechanisms of Probiotics Action in the Prevention and Treatment of Colorectal Cancer. Nutrients.

[124] Zeng H., Umar S., Rust B., Lazarova D., Bordonaro M. (2019). Secondary Bile Acids and Short Chain Fatty Acids in the Colon: A Focus on Colonic Microbiome, Cell Proliferation, Inflammation, and Cancer. Int. J. Mol. Sci..

[125] Deepak V., Sundar W.A., Pandian S.R.K., Sivasubramaniam S.D., Hariharan N., Sundar K. (2021). Exopolysaccharides from *Lactobacillus acidophilus* Modulates the Antioxidant Status of 1,2–Dimethyl Hydrazine-Induced Colon Cancer Rat Model. 3 Biotech.

[126] Clapp M., Aurora N., Herrera L., Bhatia M., Wilen E., Wakefield S. (2017). Gut Microbiota’s Effect on Mental Health: The Gut-Brain Axis. Clin. Pract..

[127] Chao L., Liu C., Sutthawongwadee S., Li Y., Lv W., Chen W., Yu L., Zhou J., Guo A., Li Z. (2020). Effects of Probiotics on Depressive or Anxiety Variables in Healthy Participants Under Stress Conditions or With a Depressive or Anxiety Diagnosis: A Meta-Analysis of Randomized Controlled Trials. Front. Neurol..

[128] Ma T., Jin H., Kwok L.-Y., Sun Z., Liong M.-T., Zhang H. (2021). Probiotic Consumption Relieved Human Stress and Anxiety Symptoms Possibly via Modulating the Neuroactive Potential of the Gut Microbiota. Neurobiol. Stress.

[129] Tette F.-M., Kwofie S.K., Wilson M.D. (2022). Therapeutic Anti-Depressant Potential of Microbial GABA Produced by *Lactobacillus rhamnosus* Strains for GABAergic Signaling Restoration and Inhibition of Addiction-Induced HPA Axis Hyperactivity. Curr. Issues Mol. Biol..

[130] Den H., Dong X., Chen M., Zou Z. (2020). Efficacy of Probiotics on Cognition, and Biomarkers of Inflammation and Oxidative Stress in Adults with Alzheimer’s Disease or Mild Cognitive Impairment—A Meta-Analysis of Randomized Controlled Trials. Aging.

[131] Silva Y.P., Bernardi A., Frozza R.L. (2020). The Role of Short-Chain Fatty Acids from Gut Microbiota in Gut-Brain Communication. Front. Endocrinol..

[132] Martínez-Guardado I., Arboleya S., Grijota F.J., Kaliszewska A., Gueimonde M., Arias N. (2022). The Therapeutic Role of Exercise and Probiotics in Stressful Brain Conditions. Int. J. Mol. Sci..

[133] Li T., Chu C., Yu L., Zhai Q., Wang S., Zhao J., Zhang H., Chen W., Tian F. (2022). Neuroprotective Effects of Bifidobacterium Breve CCFM1067 in MPTP-Induced Mouse Models of Parkinson’s Disease. Nutrients.

[134] Abd Mutalib N., Syed Mohamad S.A., Jusril N.A., Hasbullah N.I., Mohd Amin M.C.I., Ismail N.H. (2023). Lactic Acid Bacteria (LAB) and Neuroprotection, What Is New? An Up-To-Date Systematic Review. Pharmaceuticals.

[135] Dantzer R. (2018). Neuroimmune Interactions: From the Brain to the Immune System and Vice Versa. Physiol. Rev..

[136] Ullah H., Arbab S., Tian Y., Liu C., Chen Y., Qijie L., Khan M.I.U., Hassan I.U., Li K. (2023). The Gut Microbiota–Brain Axis in Neurological Disorder. Front. Neurosci..

[137] He J., Chang L., Zhang L., Wu W., Zhuo D. (2023). Effect of Probiotic Supplementation on Cognition and Depressive Symptoms in Patients with Depression: A Systematic Review and Meta-Analysis. Medicine.

[138] Papalini S., Michels F., Kohn N., Wegman J., van Hemert S., Roelofs K., Arias-Vasquez A., Aarts E. (2019). Stress Matters: Randomized Controlled Trial on the Effect of Probiotics on Neurocognition. Neurobiol. Stress.

[139] Zawistowska-Rojek A., Zaręba T., Tyski S. (2022). Microbiological Testing of Probiotic Preparations. Int. J. Environ. Res. Public Health.

[140] Ren H., Zentek J., Vahjen W. (2019). Optimization of Production Parameters for Probiotic *Lactobacillus* Strains as Feed Additive. Molecules.

[141] Afzaal M., Saeed F., Hussain M., Ismail Z., Siddeeg A., AL-Farga A., Aljobair M.O. (2022). Influence of Encapsulation on the Survival of Probiotics in Food Matrix under Simulated Stress Conditions. Saudi J. Biol. Sci..

[142] Martín R., Langella P. (2019). Emerging Health Concepts in the Probiotics Field: Streamlining the Definitions. Front. Microbiol..

[143] Abouelela M.E., Helmy Y.A. (2024). Next-Generation Probiotics as Novel Therapeutics for Improving Human Health: Current Trends and Future Perspectives. Microorganisms.

[144] Vinderola G., Sanders M.E., Salminen S. (2022). The Concept of Postbiotics. Foods.

[145] Ma L., Tu H., Chen T. (2023). Postbiotics in Human Health: A Narrative Review. Nutrients.

[146] Salminen S., Collado M.C., Endo A., Hill C., Lebeer S., Quigley E.M.M., Sanders M.E., Shamir R., Swann J.R., Szajewska H. (2021). The International Scientific Association of Probiotics and Prebiotics (ISAPP) Consensus Statement on the Definition and Scope of Postbiotics. Nat. Rev. Gastroenterol. Hepatol..

[147] Widyastuti Y., Febrisiantosa A., Tidona F. (2021). Health-Promoting Properties of Lactobacilli in Fermented Dairy Products. Front. Microbiol..

[148] Wegh C.A.M., Geerlings S.Y., Knol J., Roeselers G., Belzer C. (2019). Postbiotics and Their Potential Applications in Early Life Nutrition and Beyond. Int. J. Mol. Sci..

[149] Calabrese F.M., Ameur H., Nikoloudaki O., Celano G., Vacca M., Junior W.J., Manzari C., Vertè F., Di Cagno R., Pesole G. (2022). Metabolic Framework of Spontaneous and Synthetic Sourdough Metacommunities to Reveal Microbial Players Responsible for Resilience and Performance. Microbiome.

[150] Johns N.I., Blazejewski T., Gomes A.L., Wang H.H. (2016). Principles for Designing Synthetic Microbial Communities. Curr. Opin. Microbiol..

[151] Waller K.M.J., Leong R.W., Paramsothy S. (2022). An Update on Fecal Microbiota Transplantation for the Treatment of Gastrointestinal Diseases. J. Gastroenterol. Hepatol..

[152] Friedman J., Higgins L.M., Gore J. (2017). Community Structure Follows Simple Assembly Rules in Microbial Microcosms. Nat. Ecol. Evol..

